# Structural alterations as a predictor of depression – a 7-Tesla MRI-based multidimensional approach

**DOI:** 10.1038/s41380-024-02854-5

**Published:** 2024-11-29

**Authors:** Gereon J. Schnellbächer, Ravichandran Rajkumar, Tanja Veselinović, Shukti Ramkiran, Jana Hagen, Maria Collee, N. Jon Shah, Irene Neuner

**Affiliations:** 1 Department of Psychiatry, Psychotherapy and Psychosomatics, RWTH Aachen University, Aachen, Germany; 2 Institute of Neuroscience and Medicine 4, INM-4, Forschungszentrum Jülich, Germany; 3 JARA-BRAIN, Aachen, Germany; 4 Department of Neurology, RWTH Aachen University, Aachen, Germany; 5 Institute of Neuroscience and Medicine 11, INM-11, Forschungszentrum Jülich, Germany

**Keywords:** Depression, Neuroscience

## Abstract

Major depressive disorder (MDD) is a debilitating condition that is associated with changes in the default-mode network (DMN). Commonly reported features include alterations in gray matter volume (GMV), cortical thickness (CoT), and gyrification. A comprehensive examination of these variables using ultra-high field strength MRI and machine learning methods may lead to novel insights into the pathophysiology of depression and help develop a more personalized therapy. Cerebral images were obtained from 41 patients with confirmed MDD and 41 healthy controls, matched for age and gender, using a 7-T-MRI. DMN parcellation followed the Schaefer 600 Atlas. Based on the results of a mixed-model repeated measures analysis, a support vector machine (SVM) calculation followed by leave-one-out cross-validation determined the predictive ability of structural features for the presence of MDD. A consecutive permutation procedure identified which areas contributed to the classification results. Correlating changes in those areas with BDI-II and AMDP scores added an explanatory aspect to this study. CoT did not delineate relevant changes in the mixed model and was excluded from further analysis. The SVM achieved a good prediction accuracy of 0.76 using gyrification data. GMV was not a viable predictor for disease presence, however, it correlated in the left parahippocampal gyrus with disease severity as measured by the BDI-II. Structural data of the DMN may therefore contain the necessary information to predict the presence of MDD. However, there may be inherent challenges with predicting disease course or treatment response due to high GMV variance and the static character of gyrification. Further improvements in data acquisition and analysis may help to overcome these difficulties.

## Introduction

Major depressive disorder (MDD) is a debilitating condition with complex origins, and its development has been linked to various cerebral regions and brain networks [1]. Improving our ability of treating this disease is of high socioeconomic interest since MDD is a leading cause of disability [2]. However, major advances in the effectiveness of pharmaceutical therapy have remained elusive for many decades. This emphasizes the need for precision medicine that administers the medication type with the highest likelihood of success in each individual case. Such a prediction of disease course needs to be informed by variables that reflect on the underlying pathological process. One of the most intuitive candidates for such a variable is the structural data of the individual brain. Differences in structural disruption may reflect on subtle differences in pathophysiology that influence not only the course of disease but also what kind of therapy is most effective [3]. High-resolution images as acquired through structural MRI may contain information enabling a machine learning algorithm to predict the outcome of therapy [4]. However, first, there needs to be an estimation of how much variance is actually contained in structural MRI data. The variance present in the contrast between a patient and a healthy control could be a viable basis for such an estimate.

Simply scanning the entire brain and using all the measured values for predicting disease presence might not be advisable. In order to reduce the risk of overfitting and increase the generalizability of the results of a machine learning calculation, it is necessary to pre-select the information provided to the algorithm [5]. Since it is the question of persistence or regredience of symptoms that lies at the heart of the described endeavors, it might be prudent to focus on structures with a clear association to relevant and distinct symptomatology. One of the most notable networks implicated in MDD is the default-mode network (DMN) [6], which is known for being active during relaxed, undirected mental activity [7]. In MDD patients, altered connectivity in the DMN is associated with a tendency of rumination that prevents a state of calm relaxation [8]. Fittingly, prior research by both regular [9–11] and ultra-high [12, 13] field strength MRI has shown abnormalities in the structural features of the DMN. Predicting the presence or absence of MDD based on the structures of this network might be a first step towards using MRI data in the determination of an individual therapy approach. For our analysis, we used the Schaefer Atlas 600, which divides the brain into 600 different areas that are assigned to seventeen different networks, one of which is the DMN [14, 15]. Since this atlas allows for the fine-grained analysis of both volume and surface parameters it is particularly suited for this investigation.

Equally important to the question of which regions to investigate is the determination of the structural features to be included in the machine learning calculation. These features should reflect on both dynamic processes and stable predisposing factors to contain a maximum of relevant information. To this end, we examined gray matter volume (GMV), cortical thickness (CoT), and gyrification [16, 17]. Reductions in GMV are known to occur in MDD patients [9, 18] and CoT abnormalities have been identified in neurodegenerative diseases, schizophrenia, dissociative seizures, and depression [19–22]. Both features may be influenced by symptom severity and have thus a somewhat dynamic character [18, 23]. Gyrification helps to increase surface area and connectivity and may be a marker for a vulnerability to suffering from psychiatric diseases [5, 17, 24]. It has been proposed that gyrification is strongly influenced by neurodevelopmental and genetic factors [25], suggesting a certain degree of stability that does not change during the course of the disease. Thus, these three features should complement each other and contain the necessary data to inform about disease presence and activity.

Structural images were acquired from MDD patients and healthy participants using ultra-high field 7-Tesla-MRI to ascertain fine-grained, comprehensive data. The high field strength allows for an improved image resolution, contrast ratio, and signal-to-noise ratio (SNR) [26, 27]. These factors led to better detection of pathologic changes in various other fields like chronic inflammatory processes, ischemia, or dementia [28–31]. This more accurate characterization of cerebral pathologies contributes to the novelty of this investigation [32]. Analysis was conducted using a support vector machine (SVM), which constitutes a machine learning classifying algorithm very well suited for high dimensional data sets [33]. Prior knowledge about the DMN and its role in depressive pathophysiology determined the first step of feature extraction from the Schaefer Atlas [34]. To further reduce noise in the data, only those areas that delineated significant results in an initial statistical investigation were included. In order to add an explanatory aspect to the predictive character of this study a multiple regression analysis was used to correlate the structural data important for the classification model with disease severity.

We hypothesize that the chosen combination of ultra-high-resolution structural features, which reflect on both the volume and surface parameters of DMN regions, will enable the SVM to achieve good predictive accuracy. This is to our knowledge the first attempt to investigate the DMN using both ultra-high field strength MRI and machine learning techniques. Its results will inform on the predictive capabilities of structural MRI in the context of depressive disease.

## Methods

Forty-one MDD patients treated at the Universitätsklinik Aachen (mean age = 32.07, standard deviation = 12.00, 18 females) were matched for age and gender with forty-one healthy controls (mean age = 29.00, standard deviation = 10.52, 15 females) and recruited for our study. There were no significant age or gender differences between groups (age: *t*-test with *p* = 0.221, gender: chi-square-test with *p* = 0.499). Similar sample sizes have been successfully used for comparable analyses in the previous literature [33]. Patients currently suffering from an MDD with psychotic symptoms according to the ICD-10 and DSM-V criteria were excluded from the analysis. Healthy controls had no current or lifetime neurologic or psychiatric diseases according to the M.I.N.I. [35], and had no history of concussion or head injury. Handedness was determined using the Edinburgh Handedness Inventory [36]. Only right-handed participants were included. Qualified study personnel evaluated the described diagnostic criteria. All volunteers received financial compensation for their participation and additional travel expenses, if applicable. Patients or controls with MRI contraindications or unstable medical conditions on the day of the scan were excluded.

Depressive symptomology was measured with the Beck Depression Inventory (BDI-II; range 0–63) [37] and the Arbeitsgemeinschaft für Methodik und Dokumentation in der Psychiatrie (AMDP) system. The AMDP was developed for the objective monitoring of psychiatric therapy and is mainly used in German-speaking countries [38]. Assessments took place within a week of the MRI scan. All participants gave fully informed written consent prior to the investigation. This protocol was approved by the local Ethics Committee of the Universitätsklinik Aachen. All methods were performed in accordance with the relevant guidelines and regulations.

### MRI acquisition

MRI data acquisition was performed with a 7 T Magnetom Terra scanner (Siemens Healthineers, Erlangen, Germany) equipped with a 1Tx 32Rx Head Coil 7 T Clinical from Nova Medical (Wilmington, MA, USA) at the Institute of Neuroscience and Medicine-4 (INM-4), Forschungszentrum Jülich. MP2RAGE is a variation of the standard magnetization-prepared rapid gradient echo (MPRAGE) sequence. It acquires two gradient echo images with different inversion time (TI) and flip angle (FA) (inversion image 1 (INV1) TI = 840 ms, flip, FA = 5°, INV2 TI = 2370 ms, FA = 6°). The other sequence-related parameters were similar for both gradient echo images: echo time (TE) = 1.99 ms; repetition time (TR) = 4500 ms for SNR optimization. The image matrix was set to 320 × 300 with a 0.75 mm isotropic resolution in 208 sagittal slices. The T1 weighted anatomical images referred to here were produced by combining the two gradient echo images by means of a ratio, as explained by Marques et al. [39]. The combined image was largely free from proton density contrast, T2^*^ contrast, reception bias field, and first-order transmit field inhomogeneity.

### Structural MR data preprocessing

The raw DICOM scans were converted to 3D T1 weighted Neuroimaging Informatics Technology Initiative (NIfTI) format using MRIcron software (https://www.nitrc.org/projects/mricron). The 3D T1 weighted images were visually audited to check for poor scan quality, artifacts, and abnormal tissues using FSL View software (https://fsl.fmrib.ox.ac.uk/fsl/fslwiki/FslView). In the next step, VBM was performed using CAT12.8 (version 1907) (http://www.neuro.uni-jena.de/cat/index.html#VBM), which is designed to work with the Statistical Parametric Mapping (SPM12) toolbox (https://www.fil.ion.ucl.ac.uk/spm/software/spm12/) and MATLAB (version 9.13 (R2022b)). VBM preprocessing was performed using CAT12.8 with default settings, which included correction for bias field inhomogeneities and skull stripping followed by SPM12 affine registration. The 3D T1 weighted structural images were segmented into three voxel classes: gray matter, white matter, and cerebrospinal fluid. The GMVs and surface parameters of the DMN were calculated with the Schaefer Atlas 600 [14], and 116 GMV and surface areas were identified for further analysis. These areas are distributed among the following regions: inferior parietal lobule, dorsal prefrontal cortex, medial prefrontal cortex, ventral prefrontal cortex, lateral prefrontal cortex, precuneus posterior cingulate cortex, retrosplenial, anterior temporal, temporal, and parahippocampal cortex. The mean value of gray matter voxels within the subregions was computed for each region of interest in the native space of each subject. Following the voxel-based processing, the surface-based processing steps were performed using CAT12.8 with default settings. Surface analysis employed the provided pipeline and included the measurement of gyrification and CoT. Gyrification allows for the measurement of 3-dimensional surface complexity [40], and its calculation was based on the absolute mean curvature [41]. Using a projection-based thickness method [16], the CoT estimation and reconstruction of the central surface were completed in a single step. Subsequently, the topological defects were corrected using spherical harmonics [29], followed by surface refinement, resulting in the final central surface mesh. The individual central surfaces were spatially registered to the ‘FsAverage’ template of Freesurfer using spherical mapping with minimal distortions [42]. Finally, the local thickness values were transferred onto the ‘FsAverage’ template. CoT, as well as gyrification analysis, was performed on the basis of the Schaefer Atlas parcellation.

### Statistical analysis

Statistical analysis was performed using the R software version 4.4.1. Demographic and clinical data were analyzed using a chi-squared test (for gender) and an independent sample t-test (for age) with a significance threshold of *p* < 0.05. Differences in mean GMV, gyrification, and CoT within the DMN between the MDD patients and the healthy control group were calculated using a mixed-model repeated measures (MMRM) analysis using the “nlme” package [43]. Three models were defined with the features GMV, CoT, and gyrification as the dependent variables. Predictors were group affiliation, regions, age, and gender (in the case of GMV additionally total intracranial volume (TIV)). Random factors were the different subjects and areas. In the case of significant interaction between group affiliation and areas, this was followed by the calculation of intergroup contrasts in specific areas with the help of the “emmeans” function from the “emmeans” package [44]. Furthermore, a multiple linear regression analysis was performed in order to elucidate the association between BDI-II and AMDP scores with structural abnormalities in MDD patients. Included here were areas reaching at least a permutation importance of 0.01 in the SVM. Adjustments were made for TIV, age, and gender for the volume and for age and gender for the surface analyses. Corrections for multiple comparisons were performed for each feature individually using a false-discovery rate (FDR) on the *p*-value outputs.

### Machine learning

A pipeline was created consisting of the steps of data scaling and SVM on Python scikit-learn (version 1.2.2) with the help of pandas and numpy [45, 46]. As a first step, features were normalized using the Z-score for further processing. Here, data were centered to the mean and component-wise scaled to unit variance [45]. Given the relatively limited number of measurements and the high dimensionality of the data set, a careful pre-selection of variables for the best SVM performance was necessary. This was achieved by only considering those DMN areas that delineated significant contrasts in the MMRM analysis. The tuning variables in the SVM were the error penalty C, which influences the likelihood of overfitting; gamma, which represents the “spread” of the kernel [47]; and finally, the kernel type, which was chosen between default (linear), polynomial and a Gaussian kernel (radial basis function (rbf)) [48]. This kernel has the advantage of being able to separate complex data sets through a kernel trick that generalizes them to a non-linear hyperplane [49]. Parameter optimization was achieved using the Grid Search function provided by Python. In a further step, leave-one-out cross-validation was performed to minimize the impact of random effects. These steps were undertaken separately for each of the included features. Finally, a permutation feature importance was calculated for each model [50, 51] using the Python scikit-learn (version 1.2.2) toolbox with n_repeats = 10. Permutation feature importance describes the decrease in model accuracy when a specific variable is randomly shuffled [52]. This indicates how much of the SVM classification performance depends on this value.

## Results

### Statistical analysis

The MMRM analysis delineated for the interaction group*areas the following results: GMV *χ*^2^ (115) = 295.97, *p* < 0.001, CoT *χ*^2^ (115) = 101.57, *p* = 0.810, gyrification *χ*^2^ (115) = 153.79, *p* = 0.009. Contrasts between patients and controls were significant for GMV in the left inferior parietal lobule, the bilateral dorsal, medial, lateral, ventral, and left ventrolateral PFC as well as the bilateral temporal cortex and parahippocampal cortex (PHC) (Supplementary Table 1). Relevant gyrification contrasts were found in the areas of left precunes/PCC 3, right precuneus/PCC 7 and 8, medial PFC 2 and 8, left ventral PFC 6, left retrosplenial cortex 1 and right retrosplenial cortex 1. GMV areas with permutation importance >0.01 were the left Temp 4, left PHC 2, right medial PFC 1, right medial PFC 5, right anterior Temp 2, and right PHC 2 (Fig. 2D).

**Fig. 1:**
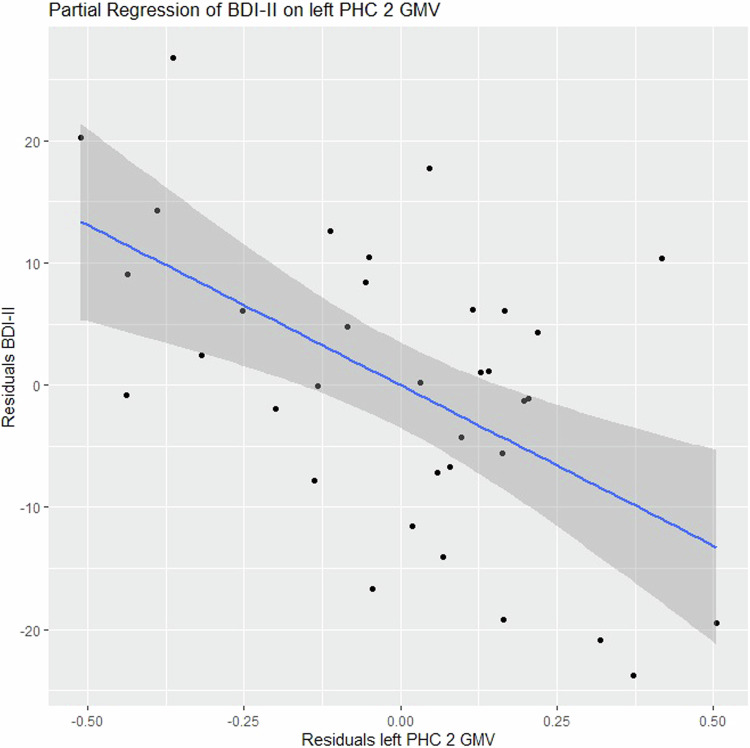
Scatter plot of a partial regression showing symptom severity correlation with the GMV of the left parahippocampal gyrus area 2 (*p*_*fdr*_ = 0.03).

Regression analysis delineated a negative correlation with BDI-II test scores in the left PHC 2 (β = −0.415, *p*_*fdr*_ = 0.03) (Figs. 1 and 3). AMDP score results did not demonstrate significant correlations in the regression analysis. Correlating the eight areas identified by the permutation procedure using the feature gyrification with BDI-II or AMDP scores delineated no significant results.

### Support vector machine

**Fig. 2:**
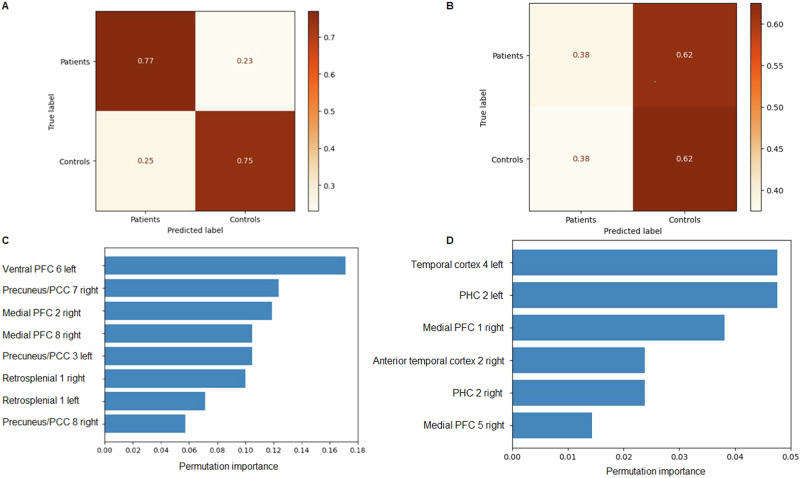
Convolution maps of the SVMs. Each field contains the ratio of correct classifications. **A** SVM based on gyrification. **B** SVM based on GMV. **C** Results of the permutation procedure of the gyrification variables. **D** Results of the permutation procedure of the GMV variables. List only represents the most significant results.

Using the GMV data the SVM (tuning parameters: C-value: 10, ɣ-value: 0.001, kernel type: rbf) reached a classifying accuracy of 0.48 (sensitivity (recall) 0.62, specificity: 0.38). Accuracy is defined here as the ratio between the number of correct predictions and the total number of predictions. The SVM using the feature gyrification (tuning parameters: C-value: 10, ɣ-value: 0.01, kernel type: rbf) delineated an accuracy of 0.76 (sensitivity (recall) 0.75, specificity: 0.77) (Fig. 2A). Permutation importance in the latter analysis was particularly marked in the ventral and medial PFC as well as the precuneus/PCC (Fig. 2C). The SVM provided with GMV data only achieved a low accuracy of 0.48 (tuning parameters: C-value: 10, ɣ-value: 0.001, kernel type: rbf) (sensitivity (recall) 0.62, specificity: 0.38) (Fig. 2B). Permutation importance was accordingly minimal with only the temporal cortex 4 and the left PHC 2 reaching values > 0.04 (Fig. 2D).

## Discussion

By using ultra-high field MRI provided gyrification data of the DMN the classifying SVM achieved good prediction accuracy. The variance was concentrated on the PFC and PCC. Volume alterations were not effective in separating patients from controls. There were, however, signs that the GMV of the left PHC correlates with symptom severity. These results elucidate both the dynamic and static characteristics of structural features during depressive disease. They also hint at the challenges associated with predicting symptom development based on structural markers.

### Predicting disease

GMV contrasts between patients and controls were significant in parts of the PFC as well as the temporal cortex and PHC. The delineated volume abnormalities are in line with the previous literature. Parahippocampal regions of unmedicated MDD patients are known to have lower volumes compared to medicated patients [53]. In studies on macaque primates, the PHC has been shown to be vital for non-navigational spatial memory [54]. Furthermore, it has been implicated in visuo-spatial processing and memory performance [55, 56], which are compromised in depressed patients [57]. Increased connectivity between the PCC and the PHC is a lasting “scar” and a hallmark of recurrent depression [58]. The temporal cortex is also subject to changes in cases of MDD [59]. Its pathophysiological connection can be seen, for example, in the effects of electroconvulsive therapy, which induces a recovery of both frontal and temporal functions in patients [60]. Additionally, dopaminergic abnormalities in the temporal cortex correlate with depression severity [61], and volume reductions in both the temporal cortex and the hippocampus can be found in suicidal patients [62]. Surprisingly, the SVM was not able to reliably predict the presence of disease based on the GMV of these regions. It only achieved a low predictive accuracy of 0.48 (Fig. 2B). Gyrification, on the other hand, predicted the presence of MDD with a good accuracy of 0.76 (Fig. 2A). This was achieved by providing the SVM with data of areas in the PCC, PFC as well as the retrosplenial cortex. According to the permutation procedure, the precuneus/PCC and the ventral as well as medial PFC were particularly important for model performance (Fig. 2C). The PFC as a whole is known to be strongly affected by MDD [63]. Gyrification changes in the ventral PFC are a biomarker of depressive disease [64], while the medial PFC plays a major role in the development of resilience against stressors. It is activated by the presence of control over a stressor and mediates a regulating effect on stress-responsive structures of the limbic system and the brain stem [65]. Additionally, the medial PFC has long been known for its role in memory and decision-making [66], and it seems to support navigation through the exploitation-exploration dilemmas of an ever-changing world [67]. Furthermore, it is highly interconnected with subcortical regions like the amygdala and the hippocampus and is important for top-down executive control [68]. Likewise, the aberrant gyrification seen in the precuneus/PCC reflects on current knowledge [69]. Taylor et al. demonstrated that normalizing connectivity between the dorsolateral PFC and the precuneus/PCC through neurofeedback leads to a mitigation of depressive symptoms [70], while increased connectivity between the PCC and the orbital frontal cortex is associated with MDD [71].

**Fig. 3:**
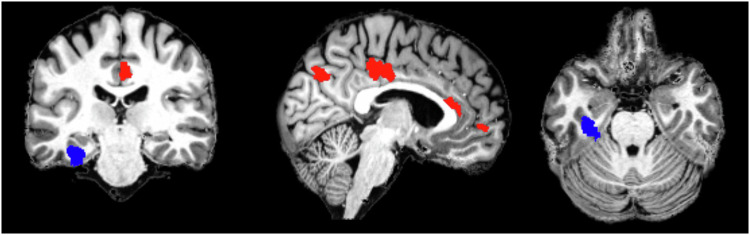
XXX. Blue: Left parahippocampal gyrus, which negatively correlated with symptom severity as measured by the BDI-II. Red: DMN areas that delineated a significant intergroup gyrification contrast in the mixed-model repeated measures analysis.

This contrast between a low predictive capability for GMV and a high one for gyrification suggests that volume might be subject to a much greater natural variance that makes a separation between diseased and healthy solely on its basis difficult. As stated in the introduction gyrification may be reflective of neurodevelopmental factors and more stable across the lifespan in comparison to GMV [72, 73]. Fittingly, our regression analysis did not delineate a significant correlation between gyrification and symptom severity in the identified areas. This emphasizes the importance of predisposing factors determined at conception or during the early neurodevelopmental years for the presence of MDD. Given the strong heritability of depression [74] this seems plausible. It is notable, that prediction did not only reach a high sensitivity but also specificity (Fig. 3A). Patients were recognized as patients with an accuracy of 0.77 and controls as controls with an accuracy of 0.75. However, in real-life conditions, there might be the danger of false positive results in the case of patients with recurring but fully remitted depression and healthy individuals who are at risk of developing MDD but never suffered from it.

Considering that the CoT measurements were not used by the SVM, due to the not significant interaction between the factors groups and areas, and that volume information did not achieve acceptable classification accuracy, only eight areas of 348 of our multidimensional data set were decisive. This shows how vitally important dimension reduction is for the future of machine learning techniques in medicine. It signals that a hypothesis-driven approach, where prior knowledge leads to a focus on only the relevant information, combined with classical statistics may be helpful even in the context of novel machine learning methods.

### Dynamic and static alterations

At the core of this investigation is the preparation of future machine learning investigations that will attempt to predict symptom development under various conditions e.g. a specific pharmacological therapy. To this end, it is useful to include also dynamic aspects in our analysis and to expand on its primarily predictive character with explanatory classical statistics. The correlation between parahippocampal GMV and symptom severity, as measured by multiple regression analysis, hints at a dynamic process influenced by or influencing the disease course. In contrast, the gyrification alterations identified by the SVM and subsequent permutation procedure were not subject to modification through disease severity and are most likely more static. These two distinct qualities of changes enable a differentiated interpretation of the role of the DMN in MDD pathophysiology.

There is an overlap between the “rich-club” of well-connected cerebral hub regions and the DMN [75, 76]. Such hubs tend to be vulnerable to pathological processes [77, 78], partly because their high level of interconnectivity leads to an increase in microstructural complexity and metabolic strain. This circumstance could be a contributing factor to our results since the only area clearly correlating with symptomatology is part of this “rich-club”. Metabolic strain implies a dynamic nature since it is subject to factors that change over time, like stress levels, nutrition, fitness, etc. [79, 80], which may contribute to the mostly episodic nature of depressive disease [81]. In our analysis, volume alterations in the left PHC correlated with disease severity as measured by the BDI-II, indicating the presence of such dynamism. Hypothalamic-pituitary-adrenocortical axis (HPA) dysregulation caused by prolonged stress leads to alterations in the PHC [82, 83] supporting this notion. Fittingly, a reduction of stress be it through physical exercise, meditation, or adequate sleep quality, often has a positive influence on disease course [84–87].

We found thus signs that both episodes of stress [88] and genetic and neurodevelopmental factors [74, 89] contribute to the development of MDD. This harmonizes well with the “multiple hit” theory often used to describe the pathogeneses of psychiatric diseases. This means that a certain predisposition for the development of pathology is inherited, but the clinical picture of MDD actually only occurs if other factors are additionally experienced by the individual. These findings emphasize the benefit of combining several morphological parameters together in one analysis since our results were able to reflect on both dynamic changes and static signs of vulnerability.

### Conclusion and limitations

Supervised machine learning methods, such as an SVM, can achieve good predictive accuracy for classifying MDD patients and healthy controls. The ultra-high resolution of the MRI images contributed to the quality and quantity of the information provided to the algorithm enabling its good results. The MMRM analysis was valuable for feature selection. In addition, regression analysis found signs of volume changes depending on symptom severity in the left PHC. Gyrification alterations in the lateral medial PFC as well as the PCC influenced the discriminatory power of the machine learning algorithm. Structural alterations in MDD have both static and dynamic characteristics. The fact that only gyrification data helped in classification may hint at future problems of predicting disease course since the relevant more dynamic aspects of brain morphology might contain a high natural degree of individual variability. Future larger data sets may help to overcome these difficulties. The relatively small study population and the benign data environment somewhat limit the interpretability of these results. The study population was relatively homogeneous and not representative of a general psychiatric patient collective. A further limiting factor is that the exact type of therapy was not considered and could have a confounding effect. Thus, it cannot be assumed that the demonstrated results can be replicated in a normal clinical environment. However, despite these factors, the results remain promising since they demonstrate that purely structural ultra-high field 7-T-MRI data already contains the necessary information to adequately discriminate between groups. Novel machine learning algorithms will increasingly support researchers in extracting information from the ever-growing fundus of neuroimaging data. The results thus encourage further machine learning-supported ultra-high field MRI investigations of morphological alterations in affective disease.

## Supplementary information

Supplementary Table 1

## Data Availability

Data will be made available on request.

## Code availability

Code will be made available on request.
