# Supplementary material for: Structural alterations as a predictor of depression – a 7-Tesla MRI-based multidimensional approach
Source: Mol Psychiatry. 2024 Nov 29;30(6):2517–24. doi: 10.1038/s41380-024-02854-5 (PMC12092240; doi:10.1038/s41380-024-02854-5)
Supplement: Supplementary file 1 — Supplementary Table 1 [file 41380_2024_2854_MOESM1_ESM.docx]

| **Gyrification** | **Grey matter volume** |
| --- | --- |
| lDefaultA_IPL_3 | l17Networks_LH_DefaultA_pCunPCC_3 |
| lDefaultA_PFCm_1 | r17Networks_RH_DefaultA_pCunPCC_7 |
| lDefaultA_PFCm_2 | r17Networks_RH_DefaultA_pCunPCC_8 |
| lDefaultA_PFCm_4 | r17Networks_RH_DefaultA_PFCm_2 |
| lDefaultA_PFCm_6 | r17Networks_RH_DefaultA_PFCm_8 |
| lDefaultB_Temp_3 | l17Networks_LH_DefaultB_PFCv_6 |
| lDefaultB_Temp_4 | r17Networks_RH_DefaultC_Rsp_1 |
| lDefaultB_Temp_5 | l17Networks_LH_DefaultC_Rsp_1 |
| vlDefaultB_PFCd_3 |  |
| lDefaultB_PFCd_4 |  |
| lDefaultB_PFCd_6 |  |
| lDefaultB_PFCl_1 |  |
| lDefaultB_PFCv_5 |  |
| lDefaultC_PHC_2 |  |
| rDefaultA_PFCm_1 |  |
| rDefaultA_PFCm_5 |  |
| rDefaultA_PFCm_7 |  |
| rDefaultB_Temp_1 |  |
| rDefaultB_AntTemp_2 |  |
| rDefaultB_PFCd_2 |  |
| rDefaultB_PFCv_5 |  |
| rDefaultC_PHC_2 |  |

**Supplementary Table 1:** Listed are all areas delineating significant intergroup contrasts in the mixed-model repeated measures analysis.
